# “I Say I'm Kind of Out”: An Insider Qualitative Study of Queer Medical Students

**DOI:** 10.1111/tct.13847

**Published:** 2024-12-22

**Authors:** Cate Goldwater Breheny, Dominic Lee, Daniel Ly, Holly Oliver, Anbreen Bi, Stephanie Bull

**Affiliations:** ^1^ Imperial College School of Medicine UK; ^2^ University of Dundee UK; ^3^ University College London UK; ^4^ University of Lincoln UK; ^5^ Medical Education Innovation & Research Centre, Department of Primary Care and Public Health, School of Public Health Imperial College London UK

**Keywords:** Diversity and Inclusion (EDI), belonging, Equality, LGBTQIA+, medical student, Queer, support

## Abstract

**Background:**

United Kingdom Queer medical students' experiences have only been explored in depth in one previous study, despite longstanding calls to address National Health Service queerphobia. The study aims to combine our participants' data with personal insights from the Queer medical student research team to both record Queer medical students' experiences and provide practical actions that can promote support, inclusivity and celebration for Queer medical students.

**Methods:**

Individual semi‐structured interviews were conducted with 12 participants across three medical schools in England and Scotland. Inductive thematic analysis was conducted. Insider insights were combined with analysis to generate practical advice for educators.

**Results:**

Participants had a broad range of Queer identities, including non‐binary, bisexual and asexual identities. Four themes were identified, as well as a table of practical advice:

1. Developing a unified Queer medical student identity: the cognitive and emotional process of aligning Queer and medical student identities;
2. A culture of discrimination: actions occurring at cultural, system and individual levels that result in negative queerphobic experiences;
3. A counterculture of support: representation and support provided by Queer faculty and doctors and Queer friends, and in curriculum material;
4. Belonging and not belonging: thoughts and feelings of acceptance and value within academic, clinical and social environments.

**Discussion:**

Queer medical students continue to experience discrimination despite calls for change. We suggest a deeper cultural reimagination of belonging as a Queer medical student, alongside practical support from educators to create this, is needed to improve Queer medical students' experiences.

## 1 Introduction

This study explores how Queer (see Box 1) medical students' gender, sexual and romantic identities affect their experiences across multiple United Kingdom (UK) medical schools. Queer medical students' experiences of discrimination [1, 2, 3, 4, 5, 6, 7, 8], and a need for change [1, 2, 5, 9, 10], are increasingly recognised. Medical educators and the institutions they work within have therefore been asked to create more inclusive learning environments for Queer medical students and ensure curriculum content about Queer healthcare is well delivered [1, 10]. However, the evidence we examined suggests improvement in medical schools has been limited to date [2, 5, 9]. For example, Queer medical students at one medical school in the UK report experiencing the curriculum as a form of violence and feeling ‘othered’ at medical school [2] and many others describe struggles to access support and mentorship [1, 6, 11]. Queer medical students do not perceive their places of study to be safe [1]. They continue to choose not to report discriminatory actions they experience and to conceal their Queer identities both actively and passively due to fear of negative consequences [3, 5, 6]. Students with some Queer identities, such as trans and non‐binary identities, may experience more discrimination and less support than others [1, 3, 9]. Overall, Queer medical students perceive levels of queerphobia to be up to 2.5 times greater than their cisgender and heterosexual peers [1, 5, 9, 12]. This lack of belonging has practical academic implications: students who are ethnically minoritised also report less supportive and often discriminatory medical school experiences and are less likely to attain as high grades as their White peers [13].

Box 1 **Statement on our choice to use the term ‘Queer’.**

LGBTQIA+ refers to people who identify as lesbian, gay, bisexual, transgender, Queer/questioning, intersex, and asexual, with the ‘+’ alluding to other diverse identities not yet represented by specific letters in the acronym. The authors, like many of the study participants, are choosing to use the term ‘Queer’ in this paper instead to refer to people who do not identify as one or more of heterosexual or cisgender or allosexual or alloromantic and who are taking a political action in reclaiming a term historically used as a slur against the LGBTQIA+ community. Several of the authors also identify as Queer. ‘Queer’ is capitalised as it is a proper noun referring to a specific community.

Queer medical students do not perceive their places of study to be safe.

We question whether this lack of progress can be attributed in part to limited understanding about the experiences of Queer medical students in the UK. To the best of our knowledge, the experiences of Queer medical students in the UK have been reported twice to date: in a single national survey performed by the British Medical Association (the main UK trade union representing British doctors and medical students), which included and combined responses from both medical students and doctors across all grades [1]; and in a qualitative study exploring the experiences of Queer medical students at one English university [2]. In‐depth studies have been conducted in the United States of America [8, 14], Canada [3, 15, 16], and Australia [17], which offer insights for UK medical educators. However, sociocultural differences in these countries means insights from additional UK medical schools across urban centres in England and Scotland are needed. There are also limited explorations of the experiences of transgender and non‐binary, and asexual, medical students in the literature to date [1, 3, 9, 18, 19, 20], which this study includes.

We further question whether another factor in the lack of positive improvement in UK Queer medical students' experiences may be the lack of practical advice for educators. This study hopes to further the development of clear guidance for educators about supporting inclusive learning environments for Queer medical students. We aim to enable this by combining the understanding gained from participants of the study with personal insights from the Queer medical study research team. As insider researchers [21, 22] the team hope to identify areas for analysis and develop practice points others may fail to consider.

This study therefore aims to combine our data, drawn from participants with a diverse range of Queer identities, with the Queer medical student research team's personal insights to describe practical actions to promote support, inclusivity and celebration for Queer medical students.

This study therefore aims […] to describe practical actions to promote support, inclusivity and celebration for Queer medical students.

## 2 Methodology

### 2.1 Study Design

We conducted an interpretivist study through which we sought to understand the significance and meaning people ascribe to experiences associated with being a Queer medical student. We developed our themes based on inductive data analysis.

### 2.2 Sampling and Context

The research was conducted in three universities across England and Scotland between October 2022 and March 2023; sampling was by convenience with the three universities representing some of the universities of the research team. Student participants were in any year of their medical degree. All interviewers were Queer medical students from different institutions to the participant, aiming to reduce the power differential and enable authentic conversation.

All interviewers were Queer medical students.

### 2.3 Reflexivity

The research team recognised they would bring their own perspectives to the research. We took care to reflect on our experiences and relative privileges and how these would shape our understanding of the experiences recounted by our participants (see Box 2). This was fundamental to our research, with Queer researchers providing specific insight and allowing deeper communication with participants as part of the in‐group. This was balanced by our supervising staff members with cisgender, heterosexual and allosexual identities who helped us identify when our own experiences were being projected onto our participants.

Box 2 **Reflexivity statements from each team member.**

CGB (they/them) is a senior medical student in London, UK, who undertook an intercalated BMedSci in Sociology and Anthropology of Medicine in Scotland, UK, during this project. They are Queer in all aspects of their identity. At medical school, they have tried to grapple with what they see as a cisheteronormative culture and curriculum and are aware that they may bring a level of cynicism from their experiences when interpreting participant narratives.

• DoL (they/them and he/him) during completion of this work was a senior medical student in Scotland, UK. They now work as a junior doctor in Scotland. They identify overarchingly as Queer; non‐binary with masculine presentation in the professional setting as well as identifying as gay and aromantic. They have unfortunately encountered discrimination whilst studying medicine and are aware that they may be primed to notice discriminatory experiences when interpreting participant narratives.

• HO (they/them) is a senior medical student in the East Midlands, UK. They are non‐binary and use the term Queer to describe their romantic and sexual identities. In the medical environment, they are frequently assumed to be a cisgender female, which means that they are regularly misgendered.

• DaL (he/him) is a senior medical student in London, UK. He is a Chinese‐Vietnamese cis man who identifies as gay or Queer. Having completed most of the medical course, he has experienced cis‐heteronormativity in both the preclinical and clinical medical school environments. His own intersectional identity means he is more likely to notice the interactions of a wider range of characteristics, such as ethnicity, disability and social economic status, when analysing participant data.

• AB (she/her) is a GP trainee who identifies as a cis‐gendered South Asian female. She reflected on her own experiences as a medical student and doctor with an intersectional identity during this process. She believes that institutional change is required and that it is essential to amplify the voices and experiences of Queer students.

• SB (she/her) is a medical educator with an interest in understanding how the educational environment impacts on students learning and wellbeing. As a White cis‐gendered heterosexual female, she kept in mind the privilege afforded through her identity and was careful to listen to the viewpoints of others.

### 2.4 Methods

DoL, CGB, DaL and HO conducted individual semi‐structured interviews online using a topic guide (see Box 3) to explore participants' views on how their gender, sexual and romantic identities affect their experiences at medical school. Participants were aware that their interviewers were Queer medical students and this was stated in the advertising calls for participants. The topic guide was used in a way that enabled participants to discuss issues pertinent to them, whilst focusing on the research aims. Interviews were audio recorded and transcribed. Each participant was given the opportunity to select a pseudonym or for the researchers to select one for them.

Box 3 **Interview topic guide used in semi‐structured interviews with participants.**

1. Can you tell us about your identity?

2. How do you feel that your Queer (replaced with terminology that participant used throughout the interview) identity interacts with your identity as a medical student?

3. Are there ways in which your Queer identity affects your experience as a medical student?

4. How represented and supported do you feel as a student with a Queer identity at medical school?

5. How do you feel that your experience compares to that of others with minoritised identities?

6. Did your Queer identity factor into your thoughts when you were applying to study medicine?

7. Have your experiences as a student with a Queer identity at medical school factored into your thoughts about your future career?

Participants were given a £20 shopping voucher in thanks for their time.

Thematic analysis was conducted according to Braun and Clarke [23]. We then combined insider perspectives with our analysis to generate practical recommendations. The analysis was inductive, with themes developed through data analysis (see Table 1).

**TABLE 1:** Table explaining our thematic analysis methodology and development of practical guidance.

Step 1: Familiarisation	Initially, the whole team immersed themselves in the data by reading the interviews multiple times. We discussed our initial impressions of the interviews across several meetings.
Step 2: Data coding	We then moved on to generating codes focusing on the unique aspects of the participants' experiences. Five researchers (CGB, DaL, DoL, AB, SB) each coded the same 3 transcripts and discussed these together. CGB, DaL, and AB then coded a further 4 transcripts each, one of which was coded by all three researchers.
Step 3: Generating initial themes	Through discussion and review of the codes, CGB, AB, DaL, and SB created a set of initial themes to capture the main insights of the data.
Step 4: Reviewing and developing themes	Initially themes were quite descriptive but CGB, AB and SB worked to identify higher level connections in the data. We referred back to the coded transcripts to ensure that the themes worked in relation to the participants experiences.
Step 5: Refining, defining and naming themes	CGB, AB and SB created a short‐written summary for each theme and all authors met to discuss these in relation to the participants experiences. This led to further expansion and collapsing of themes, refinement of the descriptors, and agreement on the theme names.
Step 6: Writing the report	CGB wrote the first draft of the thematic analysis report, which was commented on by the wider author team and refined.
Step 7: Creating practical guidance	Once thematic analysis was complete CBG, DoL and SB used their experiences as Queer medical students and a non‐Queer medical educator to develop practical actions relating to the themes identified in the study.

During this process, we had regular meetings as a whole team to justify and discuss our creation of themes. We consistently aimed to challenge one another to identify quotes and instances of our findings in our data to justify emerging themes and went through multiple iterations of themes and descriptions over several months. Having a mix of insider and outsider perspectives was crucial at this stage to ensure the themes we created were rooted in our data.

Ethical approval was granted from Imperial College London School of Medicine EERP 2022‐085, and reciprocal ethical approval was gained from an additional university in London and two universities in Scotland. Participants gave informed consent.

## 3 Results

Thirteen students were interviewed. Twelve interviews were analysed as one participant withdrew. The median interview length was 52 min (range: 37 to 70 min).

### 3.1 Participant Demographics

Four participants described not having binary gender identities, with two describing themselves as non‐binary, one as genderqueer and one as genderfluid. Five participants described themselves as women and three as men. No participants described a binary transgender identity.

Four participants described not having binary gender identities […] eight participants described attraction to multiple genders.

Eight participants described attraction to multiple genders: four described themselves as bisexual, two as bisexual/pansexual, one as bisexual/Queer, and one as Queer. Of the remaining participants, two identified as gay, one as lesbian, and one as asexual. Five participants described their romantic identity: this was congruent with their sexuality for four participants. The asexual participant was questioning their romantic orientation.

### 3.2 Participant Experiences

Four themes were constructed: 1) developing a unified Queer medical student identity; 2) a culture of discrimination; 3) a counterculture of support; and 4) belonging and not belonging.

### 3.3 Theme 1: Developing a Unified Queer Medical Student Identity

This theme describes the cognitive and emotional journey of trying to create a Queer medical student identity. This process involved how individuals develop and share their Queer identity with others and how different Queer identities lend themselves to specific, differing experiences.

All study participants discussed experiences around choosing whether and how to share their Queer identity in medical school. Participants operated a complex network of (non)disclosure across their personal and professional lives, being closeted in some situations (often with patients or with members of a clinical team) and out in others (often with Queer friendship groups).

Participants operated a complex network of (non)disclosure across their personal and professional lives

*I don't really open up anyway about that kind of stuff. ‘That kind of stuff’ [laughs] my identity, to random people. That's why I say, ‘I'm kind of out’, because if I were to fully be out then it would be something that everyone would know*. (Simon Roberts, a cisgender bisexual man and clinical years student)

Many participants chose to operate a ‘don't ask, don't tell’ policy where they displayed subtle signs of queerness such as badges, but would not share their identity openly unless asked.

*I have a name badge and it says pronouns are they/she on it, no one's commented on it. That's fine. I don't really care. It's sort of just a way for me to be like, OK. This is who I am, I'm putting it out there, but you don't have to interact with it*. (Finlay, a non‐binary preclinical student who is “bisexual or pansexual, but really I just don’t care to put a label on it”)

Some identities (bisexual, non‐binary and asexual) lent themselves to experiences of erasure – being assumed to be straight or cisgender.

*When you're dating someone of an opposite gender, it just never comes up because the assumption is, you know, that you're straight*. (Jane Anderson, a cisgender woman “somewhere between bisexual and pansexual” with a “pretty similar” romantic orientation and a preclinical student)

Asexual, non‐binary and bisexual participants found it more difficult to explain their identity to others, linked in part to their ability to control the visibility of their identity but also related to others' awareness and acceptance of that identity:

*When I came out to my friends, they didn’t understand what being asexual means at all … it feels a bit exhausting*. (Shanti, an asexual cisgender woman and preclinical student who is “heteroromantic, but not entirely sure”)

Non‐binary participants also described struggles and isolation around navigating gendered activities and spaces, including sports, social events, and clinical skills learning, which are core features of the undergraduate medical school experience.

*I just can't feel comfortable around [medical school sports teams]. Especially because they were referring to each other using gendered terms a lot because I can handle a bit of it, but not all the time*. (Ariel, a non‐binary and bisexual clinical years student)

Trying to align their medical student and Queer identities was a cognitive and emotional process for participants. Some described synergy, where their Queer identity enabled them to be a ‘better doctor’ and boosted their empathy for marginalised patients:

*I think definitely [my Queer identity has] given me a bit of a bonus in in terms of potentially caring for someone who's trans or nonbinary in the future*. (Finlay)

Others felt a tension between a professional medical identity and an ‘unprofessional’ Queer identity. This led to perceived extra pressure to be a ‘perfect’ medical student to compensate for their Queerness.

…tension between a professional medical identity and […] an ‘unprofessional’ Queer identity…

*I do think that kind of there is that thing with medicine where you just kind of want to be perfect. I think trying to be perfect, mixed with being LGBT can be quite hard sometimes because you do feel different*. (Nathan James, a homoromantic cisgender man who is “bisexual but sort of generally queer” and a clinical year student)

### 3.4 Theme 2: A Culture of Discrimination

This theme describes actions, which contribute to negative experiences for Queer medical students, which can occur on an individual level, such as direct exclusionary actions, or on a cultural or systemic level, such as cisheteronormative dominant norms around professionalism or curricular limitations and barriers to self‐expression.

Some participants described direct exclusionary actions from individuals within the educational and clinical faculty and fellow students. These ranged from feeling unheard when raising Queer issues, to hearing slurs from healthcare professionals on placement and laughter from fellow students in lectures on Queer healthcare.

Some participants described direct exclusionary actions from individuals within the educational and clinical faculty and fellow students.

*I remember one of the other [heterosexual] doctors talking about being called a dyke [as a joke by her friends] and one of her friends told her that her shoes made [her look like] like a dyke*. (Jessica Fairchild, a lesbian woman and clinical year student)

Queer medical student participants also described concern around breaking unwritten professionalism rules which they feel require them to ‘pass’ as cisgender and heterosexual.

…concern around breaking unwritten professionalism rules which they feel require them to ‘pass’ as cisgender and heterosexual.

*If you outwardly display, say, with rainbow items, that you are a member of the Queer community whilst on placement, is that going to have adverse effects in how patients treat you and how the doctors treat you and how the consultants treat you?* (Aiza, a preclinical year bisexual cisgender woman)

Participants felt excluded by curriculum content and teaching about Queer people, including a lack of coverage, superficial, unassessed coverage, or harmful coverage of Queer healthcare.

*You'll see something [in a lecture] where it says like the genders, male and female, and you'll be like, ‘that's old‐fashioned’*. (Nathan James)

### 3.5 Theme 3: A Counterculture of Support

This theme explores practices and norms pushing against a culture of discrimination, often through isolated activities, such as single teaching sessions or inclusive social events, and the actions of individual staff and students. These included representation from openly Queer faculty and senior medics, support from other Queer students and friends, and representation in the curriculum.

Participants felt supported and better understood by individual doctors, faculty members, and peers who were openly Queer in their professional lives.

*It is always useful to have a member of staff in that position who is part of the community themselves. I just think it gives them so much more nuance and understanding in issues*. (Meera, a bisexual cisgender woman who is a clinical year student)

Curriculum content that is inclusive because of the quality of the coverage, or because it is delivered well by the faculty was seen as providing representation and counteracting isolation. One participant described his experience of a lecture on trans healthcare:

Curriculum content that is inclusive […] was seen as providing representation and counteracting isolation.

*That's highlighted that maybe I am represented as a Queer person. And even though it's not something I've experienced myself, being trans or transitioning, I think that I find it positive from the faculty that they understand*. (Ashok Kumar, a cisgender gay man and clinical year student)

Friendships and networks participants had with peer members of the Queer medical student community provided support and affirmation of their own identity alongside teaching and curricular content.

*[My Queer friends are] not ‐ we don't really talk about, you know, Queer things. No, it's just usual school stress… But it's helpful to know that you're not weird*. (Shanti)

### 3.6 Theme 4: Belonging and Not Belonging

This theme describes Queer medical students' thoughts and feelings of acceptance and value within their personal lives, the university and their future life in medicine.

Participants' lives outside medical school are a backdrop to their experiences in medical school. Participants described how experiences from their home lives came with them to medical school:

*I grew up in a very homophobic family … I do think my past has made me hide who I am to these people now, because I just know how people treat you is very different*. (Simon Roberts)

Participants felt their Queer identities impacted their medical school choice, with many choosing to study in environments they perceived as more Queer‐friendly.

*It just didn’t feel that it would be as representative [of Queer people… in] places more Northern*. (Aiza)

Participants were split on whether medicine was (or should be) a particularly accepting space, or a less welcoming socially conservative environment:

*There's always the hope that doctors are… generally more liberal or accepting*. (Garath, a gay genderfluid clinical year student)

*Medical school does attract pretty conventional people, not as an insult, but as a, you know, they want a normal job. So, I think being nonbinary made me avoid my fellow medical students a bit*. (Ariel)

Participants held conflicting thoughts and feelings about their future medical life and choice of specialty, speaking positively about specialties where they felt they would be welcomed as themselves, but also feeling strongly that gender identity and sexuality should not be a barrier to specialty choice.

*[In HIV/GUM] I felt super super comfortable and just happy to be in an environment where, you know, there's loads of Queer people … And I'm considering going into surgery, but I'm a bit like “What if everyone is super, super straight and male in surgery?”* (Meera)

Participants often described ways in which they sought to manage their sense of not belonging in either their medical or Queer communities, which typically involved masking one of the two identities to others.

…they sought to manage their sense of not belonging in either their medical or Queer communities…

*I kind of was like ready to just like, pretend to be ‘normal’ [on placement]*. (Finlay)

### 3.7 Practical Advice

Combining thematic analysis with researcher insider experience generated a table of practical actions for medical educators to support Queer medical students (see Table 2).

**TABLE 2:** Table presenting our suggestions for practical actions educators can take to support Queer medical students.

What we learnt from our participants	Good practice around Queer‐inclusive attitudes and actions for medical educators, identified by participant and researcher experiences	Example of a change you might make in your day to day practice
Different Queer identities lend themselves to differing experiences at medical school with different levels of control over the visibility of their identity and identity erasure.	Avoid making assumptions that the Queer community is a monolith. Ensure you are familiar with a range of diverse Queer identities and understand some of the unique experiences that these may bring.	Ensure that there is a diverse range of identities in teaching and student support materials. Consult your library, LGBTQ+ inclusion committee or other relevant group to see if they have curated a list of resources that would support your learning about the LGBTQ community and for advice on resources that meet your needs and existing knowledge level.
Identities change over time, day to day and according to context in both the short and long term.	Accept that identity may not align with preconceptions, expectations or physical appearance.	Consider checking their pronoun badge, if they have one, or asking how students want to be referred to at that time.
Students may choose to not share their identity in some situations for a wide variety of reasons.	Appreciate that students may not always choose to share and always check with them first.	Do not disclose a student's identity on their behalf without asking their consent. At an appropriate time, you may want to ask a student why they chose not to share to give them an opportunity to discuss if they wish.
Cisheteronomative practices meant students felt excluded and not represented by medical school staff and cultures.	Listen when feelings of exclusion and concerns about teaching materials are raised and learn from these in a respectful and supportive way. Create cultural change through actions that demonstrate allyship.	Signpost your support for Queer students: introducing yourself using your pronouns, if you feel comfortable doing so, or wearing symbols such as rainbow lanyards and appreciating the meaning that this carries for members of the Queer community.
Dominant cisheteronormative cultures in medicine led to feelings of exclusion for our participants.	Promote policy that is inclusive of Queer expression and be aware of the challenges students experience from gendered and cisnormative structures and policies in medical school.	Challenge gendered norms by promoting a gender‐neutral dress code, encouraging use of pronouns in written material as an opt‐out, rather than opt‐in, system, and considering why you are using gendered groups and whether alternative ways of working, such as asking students to choose groups they are comfortable with, is a better solution.
Queer representation in curriculum content was valued by participants when delivered well, given due time and attention, and assessed.	Ensure that teaching materials on Queer healthcare are diverse, authentic, meaningful and woven throughout the curriculum. Recognise the value of working with people who have lived experience when designing teaching material, yet appreciate that the onus for your education should not sit only with them.	See if you can consult pre‐existing resources before asking someone for information that could be easily found elsewhere. When working with Queer medical students, give them information about what getting involved means and obtain their consent to have sensitive discussions before starting a project.
Direct exclusionary queerphobic actions from individuals within the faculty and from fellow students still occur and cause damage.	Increase your awareness of Queer issues, such as through engaging meaningfully with your EDI training. This will enable you to better utilise your position as an educator/clinician to step in if you hear queerphobic language or actions.	Offer students who have experienced discriminatory actions debrief opportunities as well as supporting them in raising concerns if they wish to do so. Talk about Queer patients respectfully, both in clinical practice and/or teaching materials. Avoid assumption when discussing patients.
Queer medical students have complex emotions requiring significant thought and self‐reflection around unifying their medical student and Queer identities. As a result, some participants had a sense of not belonging in their medical communities.	Create spaces for Queer medical students to share their authentic experiences, challenges and rewards openly. Build a community of support where Queer medical students, Queer faculty and Queer clinicians can connect with each other.	Support students who wish to set up flexible social spaces or medical societies, and mentorship schemes. Offer to develop learning opportunities for the whole cohort, such as Schwartz round‐style events.
Friendships and networks participants had with peer members of the Queer community provided support and affirmation of their own identity.	Champion existing Queer student‐led, community initiatives and support celebratory events such as Pride. Ask students what you can do to support them to develop student‐led initiatives in all forms, from peer teaching sessions to social events or research projects.	This could mean booking a room and sourcing snacks for a film screening or signposting to staff to support with academic activities.

## 4 Discussion

This study explores the experiences of Queer UK medical students in depth. It supports existing data, which suggest Queer medical students continue to experience discrimination in UK medical schools and globally [1, 2, 3, 14, 17]. The idea that Queer medical students experience discrimination is fundamentally not novel. This study, however, suggests discrimination continued in 2023 across several UK medical schools, despite longstanding calls and initiatives aiming to create change on an institutional level [1, 10].

…discrimination continued in 2023 across several UK medical schools, despite longstanding calls and initiatives aiming to create change…

We suggest our study shows a need for deeper, cultural change. Discriminatory cultures and exclusionary experiences mean Queer students struggle to feel a sense of belonging at medical school. When students feel like they belong, they are more able to engage and learn in a clinical placement setting [24] and have reduced levels of mental ill‐health [25]. Queer medical students experience higher levels of stress and mental ill‐health compared to cisgender and heterosexual peers [5, 8], and our participants described struggles in academic and clinical settings that may well impact on their mental health. We propose feeling that they do not belong, or that belonging is conditional on identity concealment and ‘passing’, is a key factor in Queer students' negative experiences of medical school. There is, therefore, an urgent need to consider how medical professionalism and identity can be redefined to authentically and openly welcome and celebrate Queer medical students and doctors [26].

…consider how medical professionalism […] can be redefined to authentically and openly welcome and celebrate Queer medical students…

## 5 Strengths and Limitations

Involving Queer peer researchers, who brought a unique perspective to all stages of the research process, is a strength of this work. Having three insider researchers with diverse Queer identities helped to develop a research question which included a diverse range of Queer medical students, including those historically underrepresented in research (e.g., bisexual, asexual and non‐binary identities), and recruit these students. In addition, insider researchers promoted more open dialogue with participants due to shared language and their insider identity promoting trust [22, 27, 28]. This is important in situations where participants may have historical or contextual mistrust of researcher authority, as is the case for many Queer people [22].

However, insider researchers' experiences can lead to assumptions around participants' experiences [22, 27, 28]. This was mitigated by including our two cisgender, heterosexual and alloromantic team members in the data analysis process [27]. When creating practice points, Queer members of the research team were careful not to foreground their own experiences above the research participants'. We acknowledge all experiences are unique and require equal consideration and recognition.

A further limitation was the sample size of 12. The experience of queer people should not be viewed as a monolith but a complex, intersectional and fundamentally unique experience to each individual: we were only able to capture a snapshot of our participants' experiences. However, we hope the in‐depth exploration of our data is nonetheless thought‐provoking and useful as an insight into the experiences of Queer medical students in the UK. It is worth considering also that any one person experiencing discrimination is one too many, and our data has value in aiming to prevent these discriminatory experiences.

### 5.1 Next Steps

Whilst this study explored gender diverse participants' (*n* = 4), and multiple‐gender‐attracted and asexual participants' (*n* = 9) experiences, it is still a relatively small qualitative study. It would be fruitful to repeat this study to identify if the same themes are seen with a different group of students with an emphasis to explore asexual/aromantic spectrum and trans/gender diverse medical student experiences (specifically binary trans or genderfluid experiences) further.

It may also be beneficial to continue research to develop a greater understanding of Queer students' experiences of belonging and not belonging at medical school, perhaps utilising theoretical lenses. For example, the concept of the ‘hidden curriculum’ may act as a useful lens to explore how students internalise ideas that they need to pass as cisgender and heterosexual to be professional doctors [29].

From a more practical standpoint, we would also like to explore our practice points' value. Research can explore whether these support educators to change elements of their practice, and whether this results in positive change for Queer medical students. There is scope more generally to explore what is and is not useful to support educators to build more diverse practice.

## Author Contributions

**Cate Goldwater Breheny:** conceptualization, investigation, writing – original draft, writing – review and editing, project administration, funding acquisition, methodology. **Dominic Lee:** conceptualization, investigation, funding acquisition, writing – review and editing, methodology, writing – original draft, project administration. **Daniel Ly:** conceptualization, investigation, writing – review and editing, funding acquisition, methodology. **Holly Oliver:** methodology, writing – review and editing, funding acquisition, investigation, conceptualization. **Anbreen Bi:** writing – review and editing, investigation. **Stephanie Bull:** investigation, methodology, writing – original draft, writing – review and editing, supervision.

## Conflicts of Interest

The authors declare no conflicts of interest.

## Data Availability

The data that support the findings of this study are available on request from the corresponding author. The data are not publicly available due to privacy or ethical restrictions.
